# Population pharmacokinetics of voriconazole and the role of CYP2C19 genotype on treatment optimization in pediatric patients

**DOI:** 10.1371/journal.pone.0288794

**Published:** 2023-09-11

**Authors:** Lin Hu, Shiqiong Huang, Qi Huang, Juanjuan Huang, Zeying Feng, Gefei He

**Affiliations:** 1 Department of Pharmacy, The First Hospital of Changsha, Changsha, Hunan, China; 2 Department of Pharmacy, Xiangya Hospital, Central South University, Changsha, Hunan, China; 3 Clinical Trial Institution Office, Liuzhou Hospital of Guangzhou Women and Children’s Medical Center, Liuzhou, Guangxi, China; 4 Center of Clinical Pharmacology, The Third Xiangya Hospital, Central South University, Changsha, Hunan, China; Gulu University, UGANDA

## Abstract

The aim of this study was to evaluate factors that impact on voriconazole (VRC) population pharmacokinetic (PPK) parameters and explore the optimal dosing regimen for different CYP2C19 genotypes in Chinese paediatric patients. PPK analysis was used to identify the factors contributing to the variability in VRC plasma trough concentrations. A total of 210 VRC trough concentrations from 91 paediatric patients were included in the study. The median VRC trough concentration was 1.23 mg/L (range, 0.02 to 8.58 mg/L). At the measurement of all the trough concentrations, the target range (1.0~5.5 mg/L) was achieved in 52.9% of the patients, while subtherapeutic and supratherapeutic concentrations were obtained in 40.9% and 6.2% of patients, respectively. VRC trough concentrations were adjusted for dose (C_trough_/D), with normal metabolizers (NMs) and intermediate metabolizers (IMs) having significantly lower levels than poor metabolizers (PMs) (*P*_N-P_ < 0.001, *P*_I-P_ = 0.039). A one-compartment model with first-order absorption and elimination was suitable to describe the VRC pharmacokinetic characteristics. The final model of VRC PPK analysis contained CYP2C19 phenotype as a significant covariate for clearance. Dose simulations suggested that a maintenance dose of 9 mg/kg orally or 8 mg/kg intravenously twice daily was appropriate for NMs to achieve the target concentration. A maintenance dose of 9 mg/kg orally or 5 mg/kg intravenously twice daily was appropriate for IMs. Meanwhile, PMs could use lower maintenance dose and an oral dose of 6 mg/kg twice daily or an intravenous dose of 5mg/kg twice daily was appropriate. To increase the probability of achieving the therapeutic range and improving efficacy, CYP2C19 phenotype can be used to predict VRC trough concentrations and guide dose adjustments in Chinese pediatric patients.

## Introduction

Invasive fungal diseases (IFDs) pose a significant threat to immunocompromised patients, especially those with pediatric hematological diseases or following hematopoietic stem cell transplantation, with a high mortality rate [1]. Epidemiological studies have shown a rising incidence of invasive fungal infections (IFIs) in children with hematological diseases [2, 3]. Voriconazole (VRC), a broad-spectrum triazole antifungal agent, is the first choice for the prevention or treatment of IFIs [4]. However, VRC has a narrow therapeutic window and significant interindividual variation in pharmacokinetics, which increases variability in treatment outcomes and makes individualized therapy important.

VRC is mainly metabolized by the cytochrome P450 2C19 (CYP2C19) enzyme in the liver [5]. CYP2C19 exhibits genetic polymorphism, with the CYP2C19*2, CYP2C19*3, and CYP2C19*17 mutations being major factors leading to variability in the expressed CYP2C19 metabolic phenotype. According to the CYP2C19 table [6], the proportions of different CYP2C19 phenotypes vary between Asians and Caucasians, which may lead to differences in required VRC dose. Weiss J et al. demonstrated that CYP2C19 genetic polymorphism accounted for nearly 50% variance in VRC apparent oral clearance [7]. VRC trough plasma concentrations are affected by various confounding factors [8]. Previous studies have determined the association of CYP2C19 genotype and VRC trough concentrations in pediatric patients [9, 10]. Furthermore, VRC trough plasma concentrations are also affected by non-genetic factors such as age, body weight, administration routes, concomitant medications, liver dysfunction and kidney insufficiency.

Previous research on VRC in children has been limited by small sample sizes or incomplete clinical data. There is a paucity of published population pharmacokinetic (PPK) data on VRC dosing in infants or children, and few studies have investigated VRC dose adjustment strategies in Chinese children. Studies by Wang have found CYP2C19 polymorphism, administration of omeprazole, and weight to be significant covariates for maximum velocity of metabolism (V_max_) in Chinese children [11]. Recently, investigators have proposed adjusting the VRC dose according to weight and CYP2C19 phenotype based on the established PPK model in American children [12].

However, the VRC dosing recommendations from the original manufacturer may not be suitable for Chinese children due to their different genetic backgrounds. A Chinese retrospective study has suggested that a dose of 5~7 mg/kg twice daily may be appropriate to achieve therapeutic range in children aged < 2 years [13]. Perhaps considering the high cost of genetic testing, most published retrospective studies have not included the influence of CYP2C19 phenotype in paediatric patients.

Therefore, we collected clinical data from pediatric patients and developed a PPK model of VRC to identify the factors that significantly impacted the pharmacokinetic (PK) parameters. Additionally, we conducted various dosing simulations based on the established final model to explore the optimal dosing regimen of VRC in different CYP2C19 genotypes. The primary objective of this study was to provide data supporting VRC dose optimization and to facilitate a greater number of pediatric patients in achieving the target plasma concentration.

## Materials and methods

### Study design

This was a retrospective and observational study. Medical records were collected in the department of pediatric hematology at Xiangya Hospital of the Central South University between 01 January 2018 and 31 December 2021. Patients included in the study were ≤ 14 years of age, had undergone measurement of steady-state VRC trough plasma concentrations and CYP2C19 genotyping during hospitalization, and had complete medical records available. Medical records were collected by searching the electronic medical record information system, and the following patient records were considered: ethnicity, IFIs diagnosis, treatment indication, treatment dose, treatment duration, administration routes, concomitant medications, VRC therapeutic drug monitoring (TDM) results, CYP2C19 phenotype, and liver and kidney function. IFIs were classified as possible, probable, or proven [14].

### Ethical approval

This retrospective study strictly followed the Helsinki Declaration, and the protocol was approved by the Institutional Review Board of Xiangya Hospital (approval number 2017121015). Written informed consent was obtained from all participants for the TDM of VRC, the measurement of CYP2C19 phenotype, and the use of their data. The identity information of all patients was coded to ensure privacy was not compromised.

### VRC administration

The initial dose of VRC was administered according to the manufacturer’s instructions. Based on the summary of product information [15], the recommended intravenous loading and maintenance doses of VRC in pediatric patients (2~14 years old) were 9 mg/kg and 8 mg/kg twice daily, respectively. The recommended oral maintenance doses of VRC were 9 mg/kg twice daily, and oral loading doses were not recommended. In our study, most patients did not use a loading dose because of oral administration. During subsequent antifungal treatment, the physician would adjust the VRC maintenance dose according to TDM, efficacy, or adverse drug reactions (ADRs).

#### Measurement of VRC trough plasma concentrations and CYP2C19 phenotype

The VRC steady-state trough plasma concentration was reached at 24 hours following an intravenous loading dose, and the first blood sample was obtained on day 3, considering the variability among patients. Without a loading dose, steady-state was considered to be reached on day 4~7 of twice-daily dosing [16]. All trough concentrations were collected 30 minutes before the next dose. VRC plasma concentration was measured using high-performance liquid chromatography (analytical range, 0.02 to 19.60 mg/L), as described in our previous publication [17]. CYP2C19 phenotype was determined by the DNA microarray chip method (BaiO^®^, Shanghai, China). Patients were classified as ultrarapid metabolizers (UMs) (CYP2C19*17/*17, CYP2C19*1/*17), normal metabolizers (NMs) (CYP2C19*1/*1), intermediate metabolizers (IMs) (CYP2C19*1/*2 or *1/*3), or poor metabolizers (PMs) (CYP2C19*2/*2, *2/*3 or *3/*3).

### Statistical analysis of the relationship between VRC trough plasma concentrations and CYP2C19 phenotype

The statistical analysis was conducted using IBM SPSS Statistics version 22.0 (IBM Corp., Armonk, NY, USA). The Hardy-Weinberg equilibrium was tested for each CYP2C19 polymorphism. A two-sided *P* value of less than 0.05 was considered statistically significant. The study used categorical variables such as age, gender, CYP2C19 phenotype, administration routes, and the use of co-administered proton pump inhibitors (PPIs) or glucocorticoids. Continuous variables included body weight, VRC trough plasma concentrations, VRC dose, and liver and kidney function indicators.

### Population pharmacokinetic modeling

The PPK analysis was conducted using NONMEM (non-linear mixed effects modeling, v7.5, ICON Development Solutions, Maryland, US). The PPK model was developed and implemented by using first-order conditional estimation method with η-ε interaction option (FOCE-I).

A one-compartment disposition model with first-order elimination was fit to analyze the pharmacokinetics of VRC following oral or intravenous administration. Since the majority of the modeling data in this study consisted of trough concentrations, the accuracy of estimating the absorption rate constant (K_a_) for VRC was limited. Additionally, K_a_ has minimal impact on the estimation of clearance (CL). Therefore, K_a_ was fixed at 1.19 h^-1^ as previously determined by Friberg et al. [18]. The distribution volume (V) and CL of VRC were characterized and estimated in our analysis.

The inter-individual variability (IIV) of PK parameters was described by an exponential error model:

$${\text{P}}_{\text{i}}={\text{P}}_{\text{tv}}*\text{exp}\;(\eta_{\text{i}})$$

where P_i_ represents the ith individual’s parameter value, and P_tv_ means the PPK parameter’s typical value. *ŋ*_i_ is a normal distribution with a mean of 0 and a variance of *ω*^*2*^.

The residual variability (RSV) was evaluated by comparing the following models:

$${\text{C}}_{\text{obs},\text{ij}}=\text{ln}\;\left({\text{C}}_{\text{pred},\text{ij}}\right)+\varepsilon$$

Where C_obs_ and C_pred_ represent the ith subject’s jth observed and predicted values, respectively. *ε* is a random variable distributed with a mean of 0 and variances of *σ*^*2*^.

### Covariate model

Age, gender, body weight, CYP2C19 phenotype, combination therapy, and liver and kidney function indicators were used as possible covariates to determine whether they explained the pharmacokinetic variability of VRC among people. The final model was built using a stepwise forward addition method followed by a backward elimination method. It was considered significant when the inclusion of a covariate caused an objective function value (OFV) reduction over 3.84 (*P* < 0.05), and its exclusion led to an increase in the OFV over 6.63 (*P* < 0.01).

Model Selection and Validation. The goodness-of-fit (GOF) was used to evaluate the adequacy of fitting. Non-parametric bootstrap analysis was used to assess the stability of the final model. Using the repeated random sample with replacement method, a total of 1000 duplicates were created.

### Model-based simulations

The estimated PPK parameters for CL, Vc, and F were used to simulate steady-state plasma trough concentrations with 1000 replicates using the final model. In this study, we conducted virtual simulations to evaluate six different dosage regimens (5, 6, 7, 8, 9, and 10 mg/kg twice daily) administered orally and intravenously in three CYP2C19 phenotypes (NMs, IMs, and PMs). The simulations were performed over a duration of 28 days. The probability of target attainments (PTA) of VRC trough concentrations was estimated for each dosing regimen. By running dose simulations under the same dosage regimen, we aimed to explore the sensitivity of PK parameters on the VRC steady-state plasma trough concentration. The recommended therapeutic range for VRC plasma concentration is 1.0–5.5 mg/L (4, 12).

## Results

### Patient characteristics

A total of 91 pediatric patients were ultimately included in the study. Fifty-eight (63.7%) were male, 33 (36.3%) were female, and all patients were Chinese. The median age and weight were 10 years old (range, 2 to 14 years old) and 31.0 kg (range, 9.5 to 85.0 kg), respectively. All patients had malignant hematological diseases. Fifty-two (57.1%) of the patients had acute lymphoblastic leukemia, and 55 (60.4%) of the patients had a lung infection. Proven, probable, and possible IFIs were reported for 6 (6.6%), 17 (18.7%), and 68 (74.7%) patients, respectively. The median days of VRC use were 15 (range, 3 to 148). Fifty-five (60.4%) patients received a coadministration of VRC and PPIs, and 44 (48.4%) patients received a coadministration of VRC and glucocorticoids. Patient characteristics are summarized in Table 1.

**Table 1:** Patient characteristics.

Characteristic	Total (N = 91)
Median age (years, [range])	10 (2–14)
<6	21 (23.1)
6–12	32 (35.2)
>12	38 (41.8)
Median body weight (kg, [range])	31.0 (9.5–85.0)
Sex male (no. [%])	58 (63.7)
CYP2C19 phenotype (no. [%])	
NM	37 (40.7)
IM	43 (47.3)
PM	11 (12.1)
IFI diagnosis (no. [%])	
Proven	6 (6.6)
Probable	17 (18.7)
Possible	68 (74.7)
Treatment indication (no. [%])	
Therapeutic	16 (17.6)
Empirical	49 (53.8)
Prophylactic	26 (28.6)
Median no of days of VRC use (median [range])	15 (3–148)
VRC TDM	
Median plasma trough concentration (mg/L, [range])	1.23 (0.02–8.58)
No. of measurements (median [range])	1 (1–21)
Underlying conditions (no. [%])	
Acute lymphoblastic leukemia	52 (57.1)
Acute myeloid leukemia	13 (14.3)
Lymphoma	11 (12.1)
Thalassemia	7 (7.7)
Aplastic anemia	3 (3.3)
Hemophilus syndrome	3 (3.3)
Myelodysplastic syndrome	1 (1.1)
Langerhans histiocytosis	1 (1.1)
Site of infection (no. [%])	
Lung	55 (60.4)
Oral mucosa	10 (11.0)
Intestine	3 (3.3)
Administration routes (no. [%])	
Oral	74 (81.3)
Intravenous	8 (8.8)
Intravenous to oral	6 (6.6)
Oral to intravenous	3 (3.3)
Concomitant medications (no. [%])	
PPIs ^a^	55 (60.4)
Glucocorticoids ^b^	44 (48.4)
Laboratory parameter (median [range]) ^c^	
ALB (g/L)	35.20 (20.30–49.00)
TBIL (μmol/L)	8.25 (2.10–105.20)
ALT (U/L)	31.70 (4.60–346.40)
AST (U/L)	31.05 (3.70–255.90)
Scr (μmol/L)	48.00 (27.00–252.00)
BUN (mmol/L)	3.36 (0.98–11.72)

^a^ The PPIs used were omeprazole (n = 15), pantoprazole (n = 34) and lansoprazole (n = 6).

^b^ The glucocorticoids used were methylprednisolone (n = 17), dexamethasone (n = 17) and prednisone (n = 10).

^c^ ALB, albumin; TBIL, total bilirubin; ALT, alanine aminotransferase; AST, aspartate transaminase; Scr, serum creatinine; BUN, blood urea nitrogen. Respective normal range at Xiangya Hospital, Central South University: ALB (35–50 g/L); TBIL (1.7~17.1μmol/L); ALT (9~50U/L); AST (15~40U/L); Scr (53.0~132.6 μmol/L); BUN (2.9–7.1 mmol/L).

### VRC dosing and trough concentrations

A total of 210 VRC trough concentrations from 91 children were measured in this study. The median number of measurements per patient was 1 (range, 1 to 19), and the median time between the start of VRC administration and the first trough concentration measurement was 4 days (range, 3 to 7 days). The median VRC trough concentration was 1.23 mg/L (range, 0.02 to 8.58 mg/L), while the average trough concentration was 1.09 mg/L. Of all the trough concentrations measured, the target range was achieved in 52.9% of patients, with subtherapeutic and supratherapeutic concentrations obtained in 40.9% and 6.2% of patients, respectively. The median duration of VRC treatment was 15 days (range, 3 to 148 days), and the majority of patients (81.3%) received oral VRC. In addition, 8 patients (8.8%) received intravenous VRC, and 6 patients who initially received intravenous VRC switched to oral VRC, while 3 patients who initially received oral VRC switched to intravenous VRC.

### CYP2C19 phenotypes

None of the patients belonged to the UM. The mutant type IM was the most commonly identified CYP2C19 phenotype (43/91 patients [47.3%]), followed by the wild-type phenotype (NM) (37/91 patients [40.7%]) and PM (11/91 patients [12.1%]). The allele frequencies of the CYP2C19*2 and CYP2C19*3 alleles were 29.2% and 6.6%, respectively. The Hardy-Weinberg equilibrium was respected for each allele (CYP2C19*2, *χ*^*2*^ = 1.42, *P* = 0.23; CYP2C19*3, *χ*^*2*^ = 0.11, *P* = 0.74).

**Fig 1:**
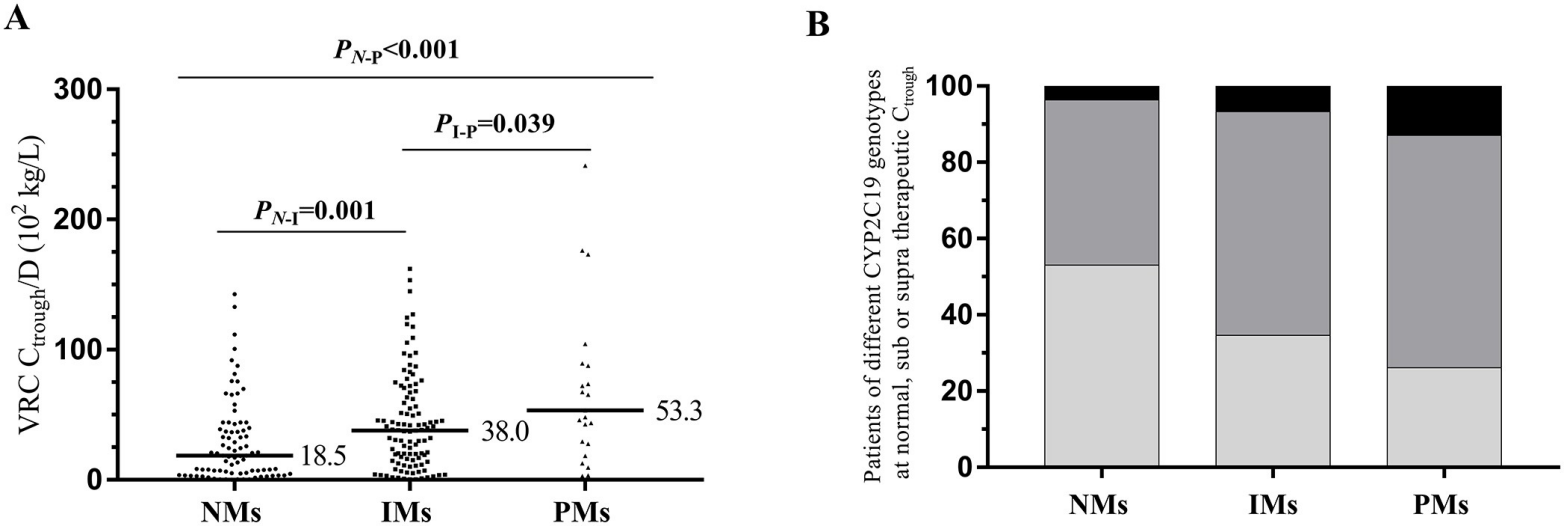
Influence of CYP2C19 phenotypes on VRC trough concentrations. (A) Comparison of VRC trough concentrations adjusted for dose (C_trough_/D) in different CYP2C19 phenotypes. Horizontal bars represent median C_trough_/D value for each CYP2C19 phenotype. *P*_N-I_: *P* value of comparison between NMs and IMs. *P*_I-P_: *P* value of comparison between IMs and PMs. *P*_N-P_: *P* value of comparison between NMs and PMs. The *P* value are indicated above the figure. (B) Percentage of patients obtaining therapeutic (dark grey), subtherapeutic (light grey) or supratherapeutic (black) concentration values in different CYP2C19 phenotypes. VRC, voriconazole. C_trough_, trough concentration.

The median VRC trough concentrations were 0.77 mg/L (range, 0.02 to 8.58 mg/L), 1.45 mg/L (range, 0.02 to 7.63 mg/L) and 2.19 mg/L (range, 0.24 to 7.66 mg/L) in NMs, IMs and PMs, respectively. The VRC trough concentrations of NMs were lower than in IMs (*P* = 0.001) and PMs (*P* = 0.001). In addition, 43.4%, 58.7% and 60.9% of VRC trough concentrations were within the target range in NMs, IMs and PMs, respectively. Furthermore, 53.0%, 34.6% and 26.1% of VRC trough concentrations were subtherapeutic in NMs, IMs and PMs, respectively. Comparison of VRC trough concentrations adjusted for dose (C_trough_/D) in different CYP2C19 phenotypes is shown in Fig 1A. Percentage of therapeutic, subtherapeutic or supratherapeutic concentration in different CYP2C19 phenotypes is shown in Fig 1B.

### Population pharmacokinetic analysis

A one-compartment model with first-order absorption and elimination was used to describe VRC pharmacokinetics. The final model included CYP2C19 phenotype as a significant covariate for CL. Table 2 presents the PK parameters of VRC for both the base and final models. The final model’s CL, Vc, and F values were 7.35 L/h, 376 L, and 52.2%, respectively. Table 2 also shows the parameter estimation and bootstrap verification of the final model. The median values of 1000 bootstraps were consistent with the respective parameters, and the PK parameters were within the 95% confidence interval (CI) in the final model. These results indicate that the model was accurate and reliable.

**Table 2:** Pharmacokinetic parameters of VRC and bootstrap results.

Parameter	Base model	Final model	Bootstrap	
	Typical value (RSE%)	Typical value (RSE%)	median	95%CI
k_a_ (h^-1^)	1.19 (fixed)	1.19 (fixed)	1.19	—
CL (L/h)	5.09 (24)	7.35 (15)	7.31	3.5–11.7
Vc (L)	532 (30)	376 (21)	393	206–854
F (%)	56.4 (24)	52.2 (15)	51.4	28.2–82.8
IM on CL	—	0.582 (12)	0.611	0.343–0.887
PM on CL	—	0.381 (14)	0.387	0.200–0.826
IIV				
ωCL	38.5 (35)	24.7 (58)	19.8	0.242–62.2
ωVc	215.6 (9)	233.2 (7)	223	148–263
Proportion residual error (%)	95.6 (12)	94.7 (9)	93.9	74.7–117.0

RSE, Relative standard error; IIV, Interindividual variability; K_a_, the absorption constant; CL, clearance; Vc, the volume of the central compartment; F, bioavailability. IM, intermediate metabolizer; PM, poor metabolizer; CI, confidence interval, 2.5th and 97.5th percentile of the ranked bootstrap parameter estimates.

### Dosing simulations

Table 3 presents the predicted VRC steady-state trough concentrations and the probability of achieving the target concentration with different dose regimens based on the final model. Among NMs, a maintenance dose of 9 mg/kg twice daily administered orally resulted in a PTA of 44.9%, while an intravenous maintenance dose of 8 mg/kg twice daily yielded a PTA of 60.9%. In the case of IMs, following the recommended oral maintenance dose of 9 mg/kg twice daily resulted in a PTA of 65.7%, whereas an intravenous maintenance dose of 5 mg/kg twice daily provided a suitable PTA of 66.1%. For PMs, an oral maintenance dose of 6 mg/kg twice daily resulted in a PTA of 64.3%, while an intravenous maintenance dose of 5 mg/kg twice daily resulted in a PTA of 56.5%. The sensitivity of CL to VRC steady-state trough concentrations exceeded 60% in three CYP2C19 phenotypes.

**Table 3:** The predicted VRC steady-state trough concentrations and the probability of achieving the target concentration with different dose regimens based on the final model.

Groups	VRC maintenance dose (mg/kg, twice daily)	Oral administration					Intravenous administration				
		Predicted concentration (mg/L)		Probability of C_trough_ attainment (%)			Predicted concentration (mg/L)		Probability of C_trough_ attainment (%)		
		mean	median	<1.0 mg/L	1.0–5.5 mg/L	>5.5 mg/L	mean	median	<1.0 mg/L	1.0–5.5 mg/L	>5.5 mg/L
NMs	5	0.64	0.50	78.1	21.9	<0.01	1.22	0.95	52.0	47.7	0.314
	6	0.77	0.60	70.9	29.1	<0.01	1.45	1.13	45.4	53.5	1.09
	7	0.90	0.71	64.6	35.4	0.027	1.70	1.32	39.7	57.9	2.41
	8	1.04	0.81	58.4	41.5	0.076	1.96	1.53	34.7	60.9	4.48
	9	1.15	0.88	54.8	44.9	0.243	2.21	1.72	30.6	62.5	6.84
	10	1.29	1.01	49.6	49.9	0.503	2.44	1.92	28.3	62.2	9.51
IMs	5	1.15	0.96	52.0	48.0	0.014	2.17	1.81	29.1	66.1	4.77
	6	1.37	1.14	44.6	55.2	0.200	2.60	2.16	24.7	65.3	9.99
	7	1.60	1.35	38.5	60.8	0.707	3.04	2.54	21.1	62.8	16.10
	8	1.82	1.52	34.1	64.1	1.82	3.46	2.89	19.3	59.4	21.40
	9	2.05	1.71	30.7	65.7	3.58	3.86	3.20	17.6	56.4	26.00
	10	2.27	1.89	27.5	66.6	5.88	4.30	3.58	16.6	52.8	30.60
PMs	5	1.85	1.38	36.9	60.9	2.27	3.48	2.61	19.4	56.5	24.10
	6	2.18	1.62	30.6	64.3	5.11	4.21	3.16	16.8	51.9	31.30
	7	2.55	1.88	25.8	63.4	10.80	4.89	3.63	14.6	49.6	35.80
	8	2.96	2.19	21.8	61.2	17.00	5.55	4.18	13.9	45.8	40.30
	9	3.32	2.48	20.1	57.7	22.20	6.19	4.65	13.1	43.5	43.40
	10	3.67	2.72	18.4	55.0	26.60	6.94	5.12	11.6	41.8	46.60

NMs, normal metabolizers; IMs, intermediate metabolizers; PMs, poor metabolizers. C_trough_, trough concentration. VRC, voriconazole.

## Discussion

At present, there are limited studies on VRC trough concentrations or PPK analysis in pediatric patients. Therefore, it is necessary to explore the PPK characteristics of VRC in children. Our study established a PPK model of VRC in pediatric hematological patients with IFIs. Our findings can provide clinicians with a reference to realize VRC dosage individualization in immunocompromised children and avoid adverse events or achieve effective VRC therapy.

To the best of our knowledge, VRC TDM is necessary due to high inter- and intraindividual variability in trough concentrations, especially in children [17]. Guidelines recommend repeated monitoring of VRC trough concentration in children. Unlike the non-linear pharmacokinetic characteristics of VRC in adults, the metabolism of VRC in children may remain linear [19].

In our final model, the typical value of VRC bioavailability was 52.2%. One possible explanation is that the absorption of VRC may differ between children and adults. Other studies also indicated that the oral bioavailability of VRC is only 66% in children aged 2~ <12 years, which is lower than 96% in adults [20]. Additionally, oral administration is more likely to have first-pass effects and drug-drug interactions. Severely ill patients receiving oral administration may have absorption and metabolism problems. The presence of CYP3A4 in the gut may lead to metabolism of VRC hence reducing the amount of available VRC for absorption. Furthermore, the absorption of oral VRC may be affected by concomitant intake of food, gastrointestinal complications, diarrhea, or other factors.

The clearance of VRC in children was approximately three times higher compared to adults [21]. A previous report indicated the clearance of VRC was 1.91 L/h in adult patients [22]. Our final PPK model demonstrated that the typical value of clearance was 7.35 L/h and the CYP2C19 genotype was the most important factor affecting VRC clearance. Our estimate was consistent with previously reported values, which also demonstrated a nearly three-fold difference compared to adult patients.

We found that the frequencies of variant alleles of CYP2C19*2 and *3 were 29.2% and 6.6%, respectively, but there was no CYP2C19*17 in our population. According to the CYP2C19 table, CYP2C19*2 and CYP2C19*3 are relatively common among East Asians (29.0% and 8.3%, respectively), while CYP2C19*17 is relatively rare (1.3%) [6]. The proportion of CYP2C19 PMs in our study was 12.1%, which was higher than the 3% reported in the French population [23] and 4% reported in an American population [24]. This indicates that the proportion of CYP2C19 PMs in the Chinese population is higher than in Europeans and Americans. As the proportion of PMs in Asian populations is higher, a lower maintenance dose may be required to achieve the same plasma concentrations. CYP2C19 phenotypes can be used to guide initial VRC dosing and often explain subtherapeutic concentrations [25]. Significant differences in VRC plasma concentrations were observed among the three CYP2C19 phenotypes in our study. PMs had significantly higher initial plasma concentrations than NMs, and other studies have also confirmed this finding. NMs have a lower likelihood of reaching the target range and are more likely to obtain subtherapeutic concentrations. Poor response to VRC therapy has been demonstrated in patients with VRC concentrations < 1.0 mg/L [26]. Therefore, it is important to pay attention to the problem of low VRC trough levels in NMs.

Takahashi et al. reported that the best probability of PTA was approximately 45% in any dose regimen with the final model of children [12]. CYP2C19 genotyping can be useful for making prompt and accurate clinical decisions, including dose adjustment or VRC discontinuation. Our research found that NMs should receive a higher dose. Dose simulations using the final PPK model indicated that a maintenance dose of 9 mg/kg orally or 8 mg/kg intravenously twice daily would be appropriate for NMs to achieve the target range. Chen et al. also found that the appropriate daily dose of VRC was as high as 20.8 mg/kg (range, 16.2~26.8 mg/kg) for NMs in Chinese pediatric patients [27]. Due to the high overall plasma concentrations in IMs and PMs, the VRC dose should be appropriately reduced to prevent adverse effects caused by excessive plasma trough concentrations. In our study, an oral maintenance dose of 9 mg/kg or an intravenous maintenance dose of 5 mg/kg twice daily was appropriate for IMs. Wang et al. suggested that the maintenance doses should decrease by about 30~40% in PMs [11]. We suggest that PMs could use a lower maintenance dose and that an oral dose of 6 mg/kg twice daily or an intravenous dose of 5 mg/kg twice daily would be appropriate for Chinese children. However, the dose adjustment strategies should be confirmed in a prospective study with patients from different racial groups.

We found that the trough concentrations of VRC in children were generally low. In our research, the average and median VRC trough concentrations were 1.09 and 1.23 mg/L, respectively. The lowest VRC trough concentration was only 0.02 mg/L. Wang et al. also found that 57.5% of Chinese children had subtherapeutic trough levels [11]. Through the PPK analysis, we identified low oral bioavailability, high clearance rate, and high proportion of CYP2C19 NM as reasons for the low VRC trough concentrations. Moreover, increased vascular permeability and the apparent distribution volume of VRC may also contribute to the decrease in plasma trough concentration in severely ill children. In such cases, initial intravenous administration of VRC is a better option for severely ill patients.

There are some limitations to our research. Our study was conducted in a single institution and only considered one genetic factor, namely CYP2C19 polymorphisms, that might influence VRC trough concentrations. However, CYP3A4 and CYP3A5 are other enzymes involved in VRC metabolism. Gautier-Veyret et al. proposed that predicting VRC plasma concentration requires considering the influence of both CYP2C19 and CYP3A genotyping [28]. We did not investigate genetic polymorphisms in CYP3A4 and CYP3A5 and could not assess their predictive value for VRC plasma concentrations in our study population.

## Conclusions

We have successfully established a PPK model of VRC among pediatric patients with hematological diseases. VRC plasma concentrations were significantly affected by CYP2C19 genetic polymorphisms. Therefore, gene-adjusted dosing can help achieve VRC trough concentration in the therapeutic range to improve efficacy and safety outcomes.

## Supporting information

S1 Raw data (XLSX)
